# Comparison of wire-guided localization (WGL) and radio-guided occult lesion localization (ROLL) in localization of non-palpable breast lesions

**DOI:** 10.1186/s12957-023-03152-0

**Published:** 2023-08-26

**Authors:** Yasmine hany elzohery, Mohammed Mohammed Gomaa, Ghada Mohamed, Waleed Mohamed Fadlalla, Sherif Nasser Taha, Maher H. Ibraheem

**Affiliations:** 1 Department of General Surgery, Faculty of Medicine, Ain Shams University, Cairo, Egypt; 2 Baheya Center for Early Detection and Treatment of Breast Cancer, Giza, Egypt; 3 Department of Diagnostic and Interventional Radiology, National Cancer Institute, Cairo University, Giza, Egypt; 4 Department of Pathology, National Cancer Institute, Cairo University, Giza, Egypt; 5 Department of Surgical Oncology, National Cancer Institute, Cairo University, Giza, Egypt

**Keywords:** Non-palpable breast lesion, Radio-guided occult lesion localization, Wire-guided localization, Breast surgery

## Abstract

**Background:**

The number of patients with non-palpable breast lesions has increased gradually. This is because of the technological development in imaging techniques and the screening programs that lead to early detection of breast lesions.

The number of patients with non-palpable breast lesions has increased gradually. This is because of the technological development in imaging techniques and the screening programs that lead to early detection of breast lesions. The aim of marking the non-palpable breast lesions is to achieve accurate lesion localization, to obtain the better cosmetic result with less tissue loss and to provide negative surgical margin.

**Aim of the study:**

In the current study, we aimed to compare the wire-guided localization (WGL) technique with the radio-guided occult lesion localization (ROLL) technique to assess their accuracy and efficacy in non-palpable breast lesions localization.

**Methods:**

This is a retrospective study conducted at Baheya center for Early Detection and Treatment of Breast Cancer from January 2018 and June2022,where 670 patients with non-palpable breast lesions underwent an excision were enrolled randomly in ROLL group (*n* = 320) and WGL (*n* = 350).

**Results:**

Both the localization time and the time of operation were significantly decreased with the ROLL in comparison to WGL(*P* < 0.001). Complete lesion excision with clear margins were reported in 119/135(88.2%) of ROLL group and in 130/159 (81.8%) of WGL group and the difference was significant (*P* < 0.001). Reoperations (re-lumpectomy or mastectomy) were done as a second procedure on 16(11.8%) of the ROLL patients compared with 29(18.2%) in the WGL patients(*P* < 0.001).

**Conclusion:**

This study shows that ROLL is as effective as WGL for non-palpable breast lesions excision. Also, ROLL improve the outcomes by decreasing the duration of surgery, localization time, achieving a higher percentage of clear margin in spite of lower specimen size and scar length.

## Introduction

The number of patients with non-palpable lesions has increased gradually. This is because of the technological development in imaging techniques and the screening programs that lead to early detection of breast lesions [1].

In early detected non-palpable lesions, breast-conserving surgery with clear safety margin is the standard surgery [2] and many procedures developed for proper non-palpable lesions localization each has advantages and risks [3].

It is necessary to accurate localize such non-palpable lesions for excision with adequate safety margin to: minimize the local recurrence and to localize the tumor bed in patient with local advanced cancer after receiving neoadjuvant chemotherapy [4].

WGL is the most common used localization method. WGL was first described by Dodd in 1965, preoperative localization by placing a wire under image guidance is the gold standard for non-palpable lesions localization since that time. Some modifications, have been made over the last 50 years such as adding a hooked tip to prevent wire migration and a reinforced portion for better identification of the lesion [5].

Established advantages of WGL are the widespread availability and the moderate price, Moreover, wires emit no ionizing radiation and can be stored safely within the imaging department but the technique has some disadvantages. The surgeon should follow the wire tip through breast tissue to reach the lesion, and this causes excessive excision of healthy tissue [6].

Wire placement technique is difficult especially in dense parenchyma, the wire should be kept in place until the operation time, this may cause discomfort and pain.The wire may be migrate, transected or displaced and wire replacement may need to be done under image guidance. Local complications during insertion may be pneumothorax [7].

The wire tip doesn’t give indication about the lesion extension and the surgeon estimates the amount of tissue to be removed intraoperative. Also, wire insertion is time consuming and is reported to be uncomfortable [8].

Radio-guided occult lesion localization [ROLL] is a recent technique, which was first described in 1998 by Luini et al. at the European Institute of Oncology, Milan, Italy [9].

In this procedure, a radioactive material is injected under image guidance in the lesion and use the gamma probe for excision [10].

Feggi et al. In 2001 described the sentinel node and occult lesion localization [SNOLL] technique, a single injection of radioactive tracer to detect the tumor and the sentinel lymph node [SLN] [11].

ROLL has gained more advantages during the last decade such as more rate of clear margins, reduction of excision volume, good cosmetic outcome, better lesion centricity in the surgical specimen and simultaneous sentinel lymph node localization. There are no serious complications related to ROLL, even though experience in the injection is needed to avoid failure of lesion identification [12].

## Aim of the study

We aimed to compare the WGL technique with the ROLL technique to assess their accuracy and efficacy in non-palpable breast lesions localization. Furthermore in our study, we compare the WGL and ROLL technique regarding complications, clear margins and reoperation rate.

## Patient and method

This retrospective study was carried out after ethical committee approval(IRB number:201910260015). Between January 2018 and June2022, 670 patients were enrolled in this study. This research was done at Baheya center for Early Detection and Treatment of Breast Cancer.

## Patient selection

All age groups were considered in this study, with a minimum age requirement of 18 years. Patients who had non-palpable lesions detected through imaging and were suitable for breast conserving surgery were eligible for enrollment. On the other hand, male patients, patients with multicentric breast cancer requiring multiple guidewires, those presenting with locally advanced disease or diffuse microcalcification, and patients with distant metastases were excluded from participation. Furthermore, pregnant and breastfeeding patients, as well as those with contraindications for breast conserving surgery and radiotherapy, or contraindications for radioisotope usage or allergies to radioisotopes, were also excluded from the study. All patients provided signed written informed consent after being informed about the study characteristics and data confidentiality. Localization of the lesion was done under stereotactic mammogram or ultrasonography guidance, on the morning of surgery day.

## Technique

**Fig. 1:**
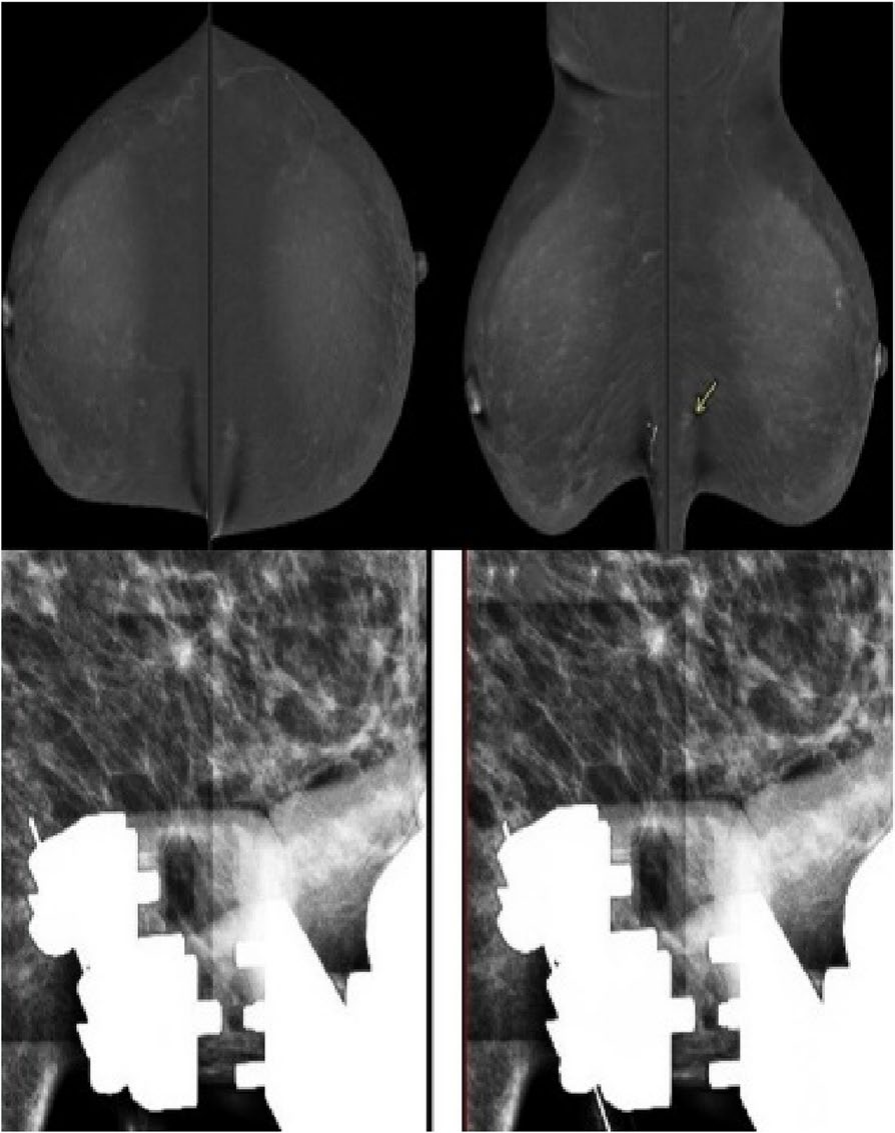
42 years old female patient came for a follow up screening which revealed a left breast lower inner quadrant inframammary deep focal asymmetry. CESM(contrast enhanced spectral mammography) was requested, which showed underlying focal non mass enhancement.The findings were considered BIRADS4, indicating the need for ROLL (radioactive occult lesion localization) and excision. A stereotactic-guided injection of radioactive technetium was administered, and post-injection imaging revealed hypodense attenuation. Following the operation, the pathology report indicated fibroadenosis with UDH (usual ductal hyperplasia) and intraductal microcalcific foci

For patients in ROLL group, injection of small amount (0.2–0.3 mL) Tc-99m- labeled colloidal human serum albumin was done in the lesion under image guidance, on the morning of surgery day Fig. 1.

**Fig. 2:**
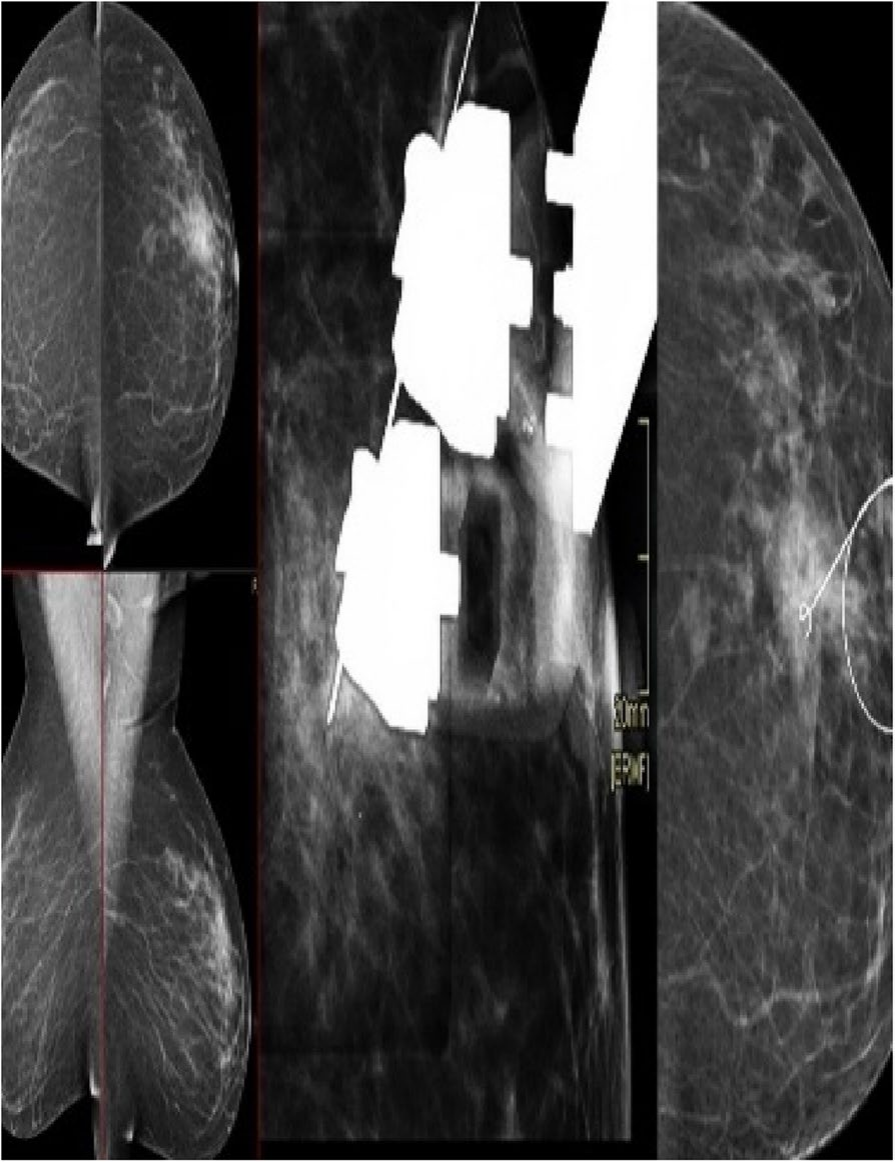
45 years old patient came for a screening and bilateral breast mammography revealed a small focal asymmetry in the upper outer quadrant (UOQ) of the left breast corresponding by ultrasound examination as an altered parenchyma. A stereotactic biopsy was performed, which revealed sclerosing adenosis warranting excision. Subsequently, a stereotactic wire localization procedure was carried out to guide the excision. The wire tip is seen within the asymmetry in the post-introduction imaging

For WGL group, the wire was preloaded in a 16–21 G needle introducer, when the tip is just beyond the lesion, the hook is deployed by fixing the needle with one hand and advancing the wire with the other. The needle is then removed over the wire. Accurate wire localization was confirmed by additional mammogram images in the CC and ML.The depth of the wire tip from the skin surface is also recorded Fig. 2.

### Sentinel node detection technique

In the ROLL group, a single injection of radiotracer was used to detect both the non-palpable lesions and sentinel lymph none in the same procedure.

## Surgical procedure

A standard conservative breast surgery was started under general anesthesia.

For ROLL, gamma probe was used to measure the radioactivity; the hotspot area with maximum gamma count rate corresponded to the site of the lesion. The incision was done over the skin site that had the highest radioactivity (hot spot) on a cosmetic basis.

After lesion excision, the cavity was checked for any residual areas of activity. Accurate lesion excision was confirmed by absence of a hot spot radioactive counts in the surgical cavity of the breast and presence of counts in the excised specimen.

For WGL, incision was made on a cosmetic basis, the surgeon followed the wire through breast tissue to reach the lesion.

SLN biopsy was performed at the same procedure with gamma probe for ROLL group while in WGL group SLN performed by injection of patent blue dye.

Radiography of the specimen was performed in all cases to confirm the presence of the non-palpable lesion in the excised specimen.

## Pathological examination

The surgical specimen was marked and put into a container with 10% neutral-buffered formalin and sent to the pathologists, the margins were stained with India ink and sliced into thin sections.

## Outcome measures

Primary outcomes for the study focused on two main factors: the adequacy of excisions with clear margins and the re-operation rate. Margins were considered "positive" if the inked margin showed gross or microscopic disease, specifically invasive carcinoma. The pathologist measured the size, volume, and weight (in grams) of the specimens.

Secondary outcomes encompassed various aspects, including patient’s satisfaction, cosmetic outcome and complications. Patient-reported outcomes were assessed through a questionnaire that measured pain and anxiety experienced during the localization technique. The scores ranged from 0 to 5, with 0 indicating no pain and 5 indicating severe pain.

Cosmetic outcomes were evaluated using a questionnaire, which assessed patient satisfaction on a scale ranging from 1 (very bad) to 5 (excellent). These assessments were conducted immediately postoperative and every six months thereafter. The difficulty of the localization technique was recorded by the radiologist, and the duration required to localize the lesions was noted in minutes.

During surgery, the duration of the procedure was recorded in minutes by the surgeons. Any complications that arose during the study were also diligently documented.

### Statistical analysis

Data were collected, tabulated and statistically analyzed using an IBM compatible personal computer with Statistical Package for the Social Sciences (SPSS) version 23. Qualitative data were expressed as Number (N), percentage (%), while quantitative data were expressed as mean, standard deviation (SD) and range (minimum–maximum). *P* value of < 0.05 was considered statistically significant.

## Results

Between January 2018 and June 2022, 670 patients were enrolled and randomized after informed consent.

The ROLL group included 320 patients with a mean age of 50 years (range 26-82years), while the WGL group included 350 patients with a mean age of 51 years (range19- 82 years).

In the ROLL group, a total of 236 females (73.8%) presented clinically with a non-palpable lesion during screening as compared to 284 patients of the WGL group (81.1%). A residual lesion after previous surgery were present in 12 females (3.8%) of ROLL patients, while 11females (3.1%) of WGL group. A residual lesion post neoadjuvant chemotherapy were present in 46 females (14.4%) of ROLL patients and 37 patients (10.6%) of WGL group. Simultaneous bilateral palpable and nonpalpable carcinoma were detected in 26 (8.1%) and 18females (5.1%),of the ROLL and WGL groups respectively Table 1.

**Table 1:** The relation of the two groups (ROLL and WGL) with different clinical, radiological and surgical parameters

variables	ROLL	WGL	*p*-value
No.of patients	320	350	
Age (year) (mean ± SD)	50.71 ± 9.72	51.67 ± 10.22	0.21
Clinical findings			
Screening	236(73.8%)	284(81.1%)	0.13
Residual lesion after previous surgery	12 (3.8%)	11(3.1%)	0.13
Residual lesion post neoadjuvant chemotherapy where clip was inserted	46(14.4%)	37 (10.6%)	0.13
patients had simultaneous bilateral palpable and nonpalpable carcinoma	26(8.1%)	18(5.1%)	0.13
Mammographic findings			
Mass	165(51.5%)	193(55.1%)	0.14
Microcalcifications	77(24.1%)	74 (21.1%)	0.14
Asymmetry and Architectural distortion	25(7.8%)	35 (10.0%)	0.14
Residual lesions post wide local excision	7 (2.2%)	11 (3.1%)	0.14
Residual distortion post neoadjuvant chemotherapy with clip inserted inside it	46(14.4%)	37 (10.6%)	0.13
Density			
ACR A	8 (2.5%)	7 (2.0%)	0.08
ACR B	279(88.6%)	283(82.5%)	0.08
ACR C	26 (8.3%)	50 (14.6%)	0.08
ACR D	2 (0.6%)	3 (0.9%)	0.08
Side			
Right	155(48.4%)	163(46.7%)	0.69
Left	161(50.3%)	179(51.3%)	0.69
Bilateral	4 (1.2%)	7 (2.0%)	0.69
Site			
UOQ	200(63.9%)	255(73.1%)	0.009
LOQ	37(11.8%)	36(10.3%)	0.009
UIQ	27 (8.6%)	32 (9.2%)	0.009
LIQ	35(11.2%)	15 (4.3%)	0.009
retroareolar	14 (4.5%)	11(3.2%)	0.009
Size /cm (mean ± SD)	1.5cm	1.7cm	< 0.001
Localization technique			
Ultrasound	264(82.5%)	267(76.3%)	0.05
Stereotactic	56(17.5%)	83(23.7%)	0.05
Localization time(min)			
Ultrasound	8 min (range 5–10 min)	18 min(range 11–20 min)	*P* < 0.001
Stereotactic	15 min (range 10–21 min)	22 min(range 20–25 min)	*P* < 0.001
Duration of surgical excision(min)			
mean ± SD	30 ± 20.3	40 ± 27.8	
median	33	45	*P* < 0.001

In the ROLL group, a total of 165 females (51.5%) presented with a non-palpable lesion showing a mass as compared to 193 patients of the WGL group (55.1%). Microcalcification were present in 77 females( 24.1%) of ROLL patients, while 74females (21.1%) of WGL group. Architectural distortions were present in 25 females (7.8%) of ROLL patients and 35 patients (10.0%) of WGL group, A residual distortion post neoadjuvant chemotherapy were present in 46 females (14.4%) of ROLL patients and 37 patients (10.6%) of WGL group.A residual lesions post wide local excision detected during post operative confirmatory imaging (satellites very close to the main excised lesion) were present in 7 females (2.2%) in the ROLL patients in comparison to 11 females (3.1%) for the WGL patients respectively Table 1.

The average tumor size in imaging was 1.5 cm in ROLL patients and 1.7 cm in WGL patients (*P* < 0.001) Table 1.

Suspicious lesions detected on imaging were mainly located in the upper-outer quadrant (200/320) in the ROLL group and (255/350) in the WGL patients.

The preoperative Tru-cut biopsy results revealed that out of 135 patients in the ROLL group and 159 patients in the WGL group, invasive carcinoma was detected. On the other hand, a benign pathology was found in 185 patients from the ROLL group and 191 patients from the WGL group.

US-guided localization was done for 264 and 267 patients of ROLL and WGL groups, respectively, whereas stereotactic technique was done for 56 and 83 patients of the ROLL and WGL group respectively.

The time of localization was significantly decreased in the ROLL as compared to WGL group (*P* < 0.001) Table 1.

Furthermore, we found a significant decrease in the duration of surgery (*p* < 0.001) in the ROLL patients (30 min, ranged15–60 min) as compared to the WGL patients (40 min, ranged 20–70 min).

Both techniques have 100% retrieval of the lesions as lesion localization and excision were successful in all cases. That was confirmed with the intraoperative imaging study of breast specimens. No significant difference was observed in the proportion of adequate excision.

We found that the removed breast specimen size was smaller in ROLL group (7.21 ± 2.90 cm) than the WGL group (7.49 ± 2.96 cm)*P* = 0.37 (Table 2).

**Table 2:** The relation of the two groups (ROLL and WGL) with different tumor size, size of resected specimen,Size of tumor/size of resected specimen ratio and agreement between radiological and surgery size

variables		ROLL	WGL	*P*-value
Size of resected tissue /cm		7.21 ± 2.9	7.49 ± 2.9	
mean ± SD		0	6	0.37
median		7	7	
Size of tumor /cm		2.04 ± 1.5	2.20 ± 1.88	0.07
mean ± SD				
Weight of resected tissue /g		39 ± 30.5	49 ± 50.6	0.001
mean ± SD				
Size of tumor/size of resected tissue ratio		0.31 ± 0.26	0.31 ± 0.26	0.43
mean ± SD		0.22	0.22	
Studied variable	Mass size by Radiology (mean ± SD)	Mass size by Surgery (mean ± SD)	Correlation coefficient	*p*-value of wilcoxon test
Roll	1.87 ± 0.98	2.04 ± 1.58	r = 0.08 *p* = 0.16	0.88
Wire	1.67 ± 0.86	2.20 ± 1.88	r = 0.2 *p* = < 0.001	< 0.001

In the ROLL patients, the diagnosis of the excised specimens was as follow: Invasive breast cancer of no special type (IBC NST)(54 females, 16.9%), ductal carcinoma in situ (DCIS) (24 female, 7.5%s), invasive tubular carcinoma (15 females,4.7%), invasive lobular carcinoma (4 females,1.2%) and 38 females had post neoadjuvant therapy status for IBC NST. In the WGL patients, IBC NST was diagnosed in 76 females (21.7%), DCIS in 35 females (10.0%), invasive tubular carcinoma in 11 females (3.1%), invasive lobular carcinoma for 9 females (2.6%) and 28 females (8.0%) had post neoadjuvant therapy status for IBC NST Table 3.

**Table 3:** Different pathologic findings as reported in the studied ROLL and WGL groups

variable	ROLL	WGL	*p*-value
Malignant	135(42.2%)	159(45.4%)	
DCIS	24(7.5%)	35(10.0%)	0.13
IBC NST	54(16.9%)	76(21.7%)	0.13
Invasive tubular carcinoma	15(4.7%)	11(3.1%)	0.13
Invasive lobular carcinoma	4(1.2%)	9(2.6%)	0.13
Post neoadjuvant therapy status for IBC NST	38(11.9%)	28(8.0%)	0.13
Benign:	185(57.8%)	191(54.5%)	
Fibroadenoma	30(9.4%)	36(10.3%)	0.13
Granulomatous lobular mastitis	2(0.6%)	0(0.0%)	0.13
Intraductal papilloma	21(6.6%)	30(8.6%)	0.13
Fibrocystic disease, scleroing adenosis, ADH& PASH	132(41.2%)	125(35.7%)	0.13

*IBC NST* Invasive breast carcinoma, No special type, *DCIS* Ductal carcinoma insitu, *ADH* Atypical ductal hyperplasia (ADH), *PASH* Pseudoangiomatous stromal hyperplasia

The number of patients diagnosed with benign pathology was 185 patients in the ROLL groups and 191 in the WGL group. Fibrocystic disease, sclerosing adenosis, intraductal papilloma, atypical ductal hyperplasia (ADH) & pseudoangiomatous stromal hyperplasia (PASH) were the most common benign findings and were detected in 132 patients of the ROLL group and 125 of the WGL group. The next most frequent benign finding was fibroadenoma, which was reported in 30 patients in the ROLL patients and 36 in the WG patients Table 3.

The number of cancer patients with involved margins differed significantly between two groups: 29 out of 159 WGL patients (18.2%) compared to 16 out of 135 ROLL patients (11.8%) (*P* < 0.001). All patients with involved margins in both groups underwent re-surgery.

Reoperations, either re-lumpectomy or mastectomy, were performed as a second procedure on 16 ROLL patients (11.8%) and 29 WGL patients (18.2%). In the WGL group, 13 out of the 29 patients with positive margins underwent wider local excision, while 16 patients underwent mastectomy. In the ROLL group, 8 patients underwent wider local excision, and 8 underwent mastectomy.

In the ROLL group, the sentinel lymph node was identified and removed in 105 patients (32.8%). Both techniques were well tolerated by patients without any major complications. However, minor complications were observed, with 12.5% of ROLL patients and 30% of WGL patients experiencing them. Specifically, 10 patients in the ROLL group had a hematoma compared to 15 patients in the WGL group and 20 ROLL patients had seroma compared to 25 patients in the WGL group.

In the WGL group,15/350,(4.2%)patients had a wire dislodge during patient’s transfer and required wire repositioning. When the wire was dislodged or displaced intraoperatively, wider tissue excision was performed. Intraoperative imaging of the specimen confirmed the presence of the non-palpable lesion, and postoperative imaging was done to detect any residual lesion and confirm removal of the clip for patients who had a wire inserted over the clip. The Pathology report was revised to indicate that the lesion was removed.

Superficial wound infection was reported in 10 ROLL patients (3.1%) and 15 WGL patients (4.2%), all of which were treated without surgery superficial wound infection was reported in 10 of the ROLL patients and 15 of the WGL patients. All these complications were treated without surgery.

Regarding cosmetic outcome, 93.7% of ROLL patients had an excellent outcome, and 6.2% had a good outcome, compared to 85.7% excellent and 14.2% good outcomes for the WGL group, with a significant difference (*P* = 0.003). As regard the scar length as measured during postoperative follow-up, showed a significant decrease in ROLL group as compared to WGL group (*p* = ***P*** < 0.001). It ranged 2.5–3 cm in ROLL patients and 4–4.5 cm in WGL patients. The site of the scar varied between direct incision on the lesion, circumareolar, or inframammary incision Table 4.

**Table 4:** The relation of both ROLL and WGL groups with the rate of positive margin, re-surgery, complication and Cosmetic outcome

variable	ROLL	WGL	*p*-value
Margin			
Involved	16 (11.8%)	29(18.2%)	*P* < 0.001
Clear	119 (88.2%)	130(81.8)	
Re-surgery			
Mastectomy	8	16	
Wider margin excision	8	13	
complication	40(12.5%)	105(30%)	< 0.001
haematoma	10(3.1%)	15(4.2%)	0.58
seroma	20(6.2%)	25(7.1%)	0.75
infection	10(3.1%)	15(4.2%)	0.58
Wire displacement		15(4.2%)	
Cosmetic outcome			
excellent	300(93.7%)	300(85.7%)	0.003
Good	20(6.2%)	50(14.2%)	
Pain	10(3.1%)	40(11.4%)	

## Discussion

The increasing use of the breast screening program has resulted in early detection of non-palpable breast lesions. Both the GWL and the ROLL technique are used to localize the impalpable lesions [13].

Precise localization is an essential step in accurate lesions excision. De Cicco et al. [14] reported that ROLL enables the surgeon to excise non-palpable lesions reliably and easily. The results in this study are the same as that study, the lesions were retrieved in the surgical specimen in all cases. Some studies that compared WGL and ROLL reported that ROLL was more precise than the WGL technique [15, 16].

In the study reported by Gennari [17], 647 patients underwent ROLL procedure, lesion was detected in 99.1% of the patients. Control imaging confirmed the presence and central localization of the lesion in 99.5% of cases.

Compared with the literature, in this study, the time of localization was significantly decreased with ROLL compared with WGL. This finding may be due to the placement of the wire as used in WGL is a technically difficult procedure and more complex than ROLL, requiring more steps, particularly in dense breast tissue in stereotactic localization.

Nadeem et al. [15] found that localization time was saving [*p* < 0.001] in ROLL. Thind et al. [18] reported that the localization time in ROLL shorter than WGL [*p* < 0.001]. The same results were found by Medina-Franco et al. [19].

In the present study, the operation time was significantly shorter in the ROLL in comparison to WGL. These findings are similar to the reported findings reported by Sajid et al and Ahmed et al [5, 20]. Also, Nadeem et al. [15] reported a significantly shorter [*p* < 0.013] procedure duration with ROLL.

This finding may be due to surgeon should follow the wire pathway, which may not be a practical route to reach the lesion. However, the gamma probe used in ROLL allow the surgeon to identify the hotspot easily and enable them to choose the shortest access route to the lesion and direct the incision and dissection more accurately, regardless of the site of radiotracer injection or the needle track and this makes the operation quicker [21].

Postma et al. [8] reported that there’s no difference in localization time or operation time and in difficulty of performing the procedure with either WGL or ROLL.

In the present study, the mean diameter of the lesions was higher in the WGL in comparison to the ROLL [2.20 ± 1.88 cm vs. 2.04 ± 1.58] with significant difference between the groups [*P* 0.07]. Studies comparing lesion sizes found that mean lesion diameter was between 1.2 cm and 1.5 cm in ROLL groups and 0.9 cm and 2.5 cm in WGL groups [18, 22].

The size of excised breast specimen did not differ significantly between the WGL group [7.49 ± 2.96 cm] and the ROLL group [7.21 ± 2.90 cm] similar to Postma study [8]. Most previous studies have not shown significant differences in excised specimen size, except Zgajnar, et al. [23] in which ROLL specimens were significantly smaller.

However, Ahmed et al reported that there was an increase in the excised tissue volume in the ROLL group [20]. However, the explanation for larger tissue volumes excision in the ROLL group is difficult to find. Potentially, a wire help the surgeon to pinpoint the center of the lesion exactly, while the maximam amount of counts [used as guidance during the ROLL procedures] is often more diffuse. So, the surgeon may continue removing additional tissue when radioactivity is still traceable within the breast.

The mean specimen weight was 39 g [range: 8–70 g] in the ROLL patients and 49 g [range: 10–150 g]in the WGL patients, with no significant difference between the groups.

Mariscal Martínez et al. [21] found that the mean specimen weight was [68.1 g] in the ROLL patients in comparison to WGL [67.3 g]. However, Rampaul et al. [16] found that the mean weight of specimen was 34 g in the ROLL patients and 31 g in the WGL patients.

About 15–20% of patients who underwent excision of occult lesion in the literature showed malignant findings, in our study, 42.2% patients in the ROLL patients and 45.4% in the WGL patients had malignant findings.

In this study, we found that ROLL is superior to WGL in the term of tumor free margins. A significant lower number 16 of ROLL group had positive margins in comparison to WGL group 29. In the study reported by Nadeem et al. [15] a larger number 43% of WGL patients had inadequate excisions as compared to 17% in the ROLL group.

Thind et al. [19] reported that 84% of ROLL patients and 60% of the WGL patients had clear surgical margins [p ¼ 0.001]. In the study reported by Zgajnar et al. [23] 70% of ROLL and 44% of the WGL patients had tumor free margins [p ¼ 0.005].

This can be explained by better lesion centricity in the ROLL allow the surgeon to excise the suspicious lesion guided by the intensity and frequency of the radioactivity, when it decreased, this mean that they are away from the center of the lesion.

A large study on ROLL reported by van Rijk et al. [24] on 368 patients found clear margins in 89% of patients and a 97% sentinel lymph node identification rate.

Gray et al. [25] found a lower rate of re-surgery with ROLL [26%] compared to WGL [57%] [*p* = 0.02]. So, the ROLL has a lower rate of re-operation for positive margins.

Sajid et al. [5] compared the WGL and ROLL in their metaanalysis of 4 studies and reported that the rate of complication was more in WGL group, no significant difference and major complication were found between the groups.

In this study, there were no major complications except for seroma, hematoma and wire displacement [as occurred in 15 patients in our study].

This study showed that pain during the localization procedure was lower in ROLL patients in comparison to WGL patients. The finding is similar to the study reported by Moreno et al. [26] and Rampaul et al. [16]. The explanation for this may be due to the localization time is longer in WGL in comparison to ROLL and difficult wire technique especially in dense breast parenchyma.

The present study is similar to other studies showed that ROLL technique has a better cosmetic result with lesser breast volume excision as it allow an esthetic incision into the skin [15, 27].

There was no report about scar length in the previous studies. But, in our study the scar length was ranged between 2.5–3 cm in the ROLL patients and 4–4.5 cm in the WGL patients. It was significantly decreased with ROLL.

According to Postma et al no significant difference in cosmesis was noted between WGL and ROLL [8].

The present study found that ROLL in the lower quadrant lesions is more accessible than WGL because the wire is transversing the breast from upper pole to the lower pole especially in case need stereotactic guided localization.

The limitation of this study is mainly the number of patients, especially patients who had localization under mammography guide. Also, this study has been conducted at a single tertiary center, a multi-centric study is required to refine and generalize the results.

## Conclusion

This study shows that ROLL is as effective as WGL for non-palpable breast lesions excision. Also, ROLL improve the outcomes by decreasing the duration of surgery, localization time, achieving a higher percentage of clear margin in spite of lower specimen size and scar length and avoid complications as wire displacement. In addition, sentinel lymph node biopsy can be performed during the same procedure. Therefore, we suggest ROLL as an effective alternative to WGL in non-palpable breast lesions localization.

## Data Availability

The datasets used and/or analyzed during the current study are available from the corresponding author on reasonable request.

## Abbreviations

ROLL: Radio-guided occult lesion localization
WGL: Wire-guided localization
